# Professional Items

**Published:** 1859-01

**Authors:** 


*Professional Items.*—Among the American dentists now in Paris, are Dr Thos. W. Evans, formerly of Lancaster, Pa., and his brother Theodore Evans; Dr. Gage, formerly of Mobile, Ala.; Dr. L. S. Parmly, of New Orleans; Dr. Horner, of Philadelphia, Mr. James Fowler and Dr. Crane, both of New York. There is also another Mr. Fowler, of the firm of Fowler & Preterre, and Dr Koth, formerly dentist to the Queen of Spain, claiming to be an American dentist.—Dr. L. S. Burridge, has, we understand, returned to Rome, and re-

*As the right to manufacture the alloy, as well as the entire process, has been secured to the inventor, Dr. A. A. Blandy, by letters patent, the exact composition may be seen in the specification which accompanied his application for the patent.

sumed his practice.—Dr. James North, it is said, has established a very lucrative practice in Vienna.—Drs. S. H. Dumont and F. P. Abbot, are at Berlin. Dr. Dumont, though a Belgian, was a pupil of Dr. Maynard's, and is a graduate of the Baltimore College of Dental Surgery.—Mr. McKeehan, said to be an American dentist, is established in practice at Madrid.—At Hamburg, Mr. Cohen, also claiming to be an American dentist, is doing a good practice.—At Stockholm, the brothers Tellander, said to have studied their profession in New York, are established in practice.—The American dentists of London are Drs. Ballard, of New York, and Mr. Rann, of Toronto, Canada West. We have been informed that each is doing a lucrative practice. —Dr. C. R. Coffin, formerly of Portland, Me., is doing a large practice at Manchester, England.—We notice by the Washington papers, that Dr. Van Camp, a successful operator, of many years standing, who, after an absence of several years from the United States, occasioned by a severe attack of typhoid fever, which left him in so feeble a state of health that a sea voyage was advised, has returned, and located himself in this city. While away, we believe he spent the greater portion of his time at the Pacific Islands, where he received from Government the appointment of U. S. Consul —Dr. H Colburn, after an absence of two years, has returned and resumed his practice in Baltimore.—We learn from a professional friend, that a thoroughly and greatly enlarged edition of Dr. James Robinson's work on the Teeth, is soon to be published in London, and probably by Messrs. Lindsay & Blakiston, of Philadelphia.—Mr. Churchhill, a London publisher, announces in press a Manual of Practical Dentistry, by Mr. Tomes.—Dr A. A. Blandy has returned to Europe, and will probably be absent from the United States, ten or twelve months.

